# Comparison of Quantification Using UV-Vis, NMR, and HPLC Methods of Retinol-Like Bakuchiol Present in Cosmetic Products

**DOI:** 10.3390/ijms26146638

**Published:** 2025-07-10

**Authors:** Matylda Grzelecka, Paweł Siudem, Natalia Tyburc, Liling Triyasmono, Ulrike Holzgrabe, Katarzyna Paradowska

**Affiliations:** 1 Department of Organic and Physical Chemistry, Faculty of Pharmacy, Medical University of Warsaw, Banacha 1, 02-097 Warsaw, Polandkatarzyna.paradowska@wum.edu.pl (K.P.); 2 Institute of Pharmacy and Food Chemistry, University of Würzburg, Am Hubland, DE-97074 Würzburg, Germany; liling.triyasmono@ulm.ac.id (L.T.); ulrike.holzgrabe@uni-wuerzburg.de (U.H.); 3 Department of Pharmacy, Faculty of Mathematics and Natural Sciences, Lambung Mangkurat University, Banjar Baru 70713, Indonesia

**Keywords:** cosmetics, bakuchiol, ^1^H qNMR, HPLC, UV-Vis

## Abstract

Retinoids are used in cosmetics as anti-aging ingredients, along with other substances. However, due to limitations in use (such as photodegradation), it seems necessary to look for retinoid alternatives to be applied in cosmetic products. Bakuchiol, a natural alternative of retinoids, isolated from *Psolarea corylifolia*, is one such compound. It has great cosmetic potential and its mechanism of action is not yet fully explored. From the point of view of the bioactive compound, it is also essential to develop a method for rapid quality control of cosmetic preparations containing bakuchiol. The aim of this study was to apply and compare methods for the quantification of bakuchiol in cosmetic products using UV-Vis, ^1^H qNMR, and HPLC. The results show the possibility of using the ^1^H NMR method in the routine quality control of cosmetics with bakuchiol because of its comparable results with HPLC analysis and significantly shorter analysis time.

## 1. Introduction

Aging is a natural and inevitable process which is largely influenced by genetic factors. Among other symptoms, it includes various changes in the skin. Skin aging results from intrinsic processes like telomerase activity as well as external factors such as UV radiation due to sun exposure and pollution, known as photo-aging [1]. Cosmeceuticals, bridging cosmetics and pharmaceuticals, target those factors to rejuvenate the skin, with vitamin A derivatives (retinoids) being particularly effective due to the activation of RAR (retinoic acid receptor) [2,3]. Retinoids, while beneficial for anti-aging, can cause adverse reactions like skin irritation and increased sensitivity to light.

**Figure 1:**
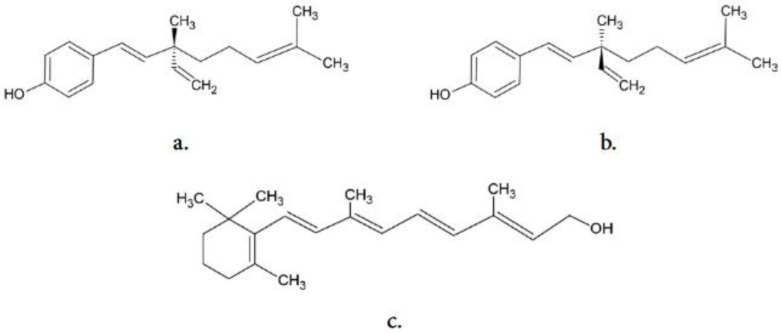
The structure of: (**a**) (S)-bakuchiol, (**b**) (R)-bakuchiol, and (**c**) all-trans retinol.

Bakuchiol (C_22_H_34_O_2_, Figure 1) is believed to be an efficient agent in the prevention of skin aging as it exhibits antioxidant activity [4] and stimulates the expression of the same genes as natural retinoids [5]. Bakuchiol was first isolated from the seeds of *Psolarea corylifolia* (*Fabaceae*) in 1966 [6]. In the same year, its complete structural configuration was established [7]. Bakuchiol is an optically active substance and the naturally occurring stereoisomer is S-(+)-bakuchiol. In 1973, the first successful biochemical synthesis of bakuchiol was carried out [8]. A natural source from which bakuchiol can be obtained is the fruit of *Psoralea corylifolia* (syn. *Cullen corylifolium*) [9]. The concentration of bakuchiol in those fruits ranges from 1% to 7% by dry weight [10]. This compound has also been isolated from roots, seeds, dry leaves, and fruits of other plant species belonging to various families [11,12,13,14,15,16,17].

The mechanism of action and all of the possible molecular targets for bakuchiol are yet to be studied [18]; however, based on the observed positive effects on the skin [19], it is frequently applied in products available on the market. Thus, methods of quality control are urgently needed. This is why, in our study, spectroscopic and chromatographic methods of qualitative and quantitative analysis were applied to preselect the most promising ones in terms of the simplicity, cost, and time of analysis.

## 2. Results

Six commercially available cosmetic formulations described as ‘face serum’ were obtained from the Polish market. The samples varied both in composition (the majority of them were oil solutions, two of them were emulsions) and in price (ranging from drug stores to ‘eco’ stores). The major ingredients are listed in Table 1.

The chief aim of this research was to develop a method for the rapid and simple identification of the active ingredient bakuchiol in cosmetic products in a direct manner. The samples were obtained from drugstores. The selection criteria comprised the popularity ranking among consumers as well as the price of the product. It was assumed that the price of the product would depend on the quality of the active substance and the form of the preparation, and hence its effectiveness, as well as the packaging. The ranking encompassed both high-end and lower-priced preparations. Six cosmetic preparations (in the form of sera) were selected for analysis.

Bakuchiol is insoluble in water but soluble in alcohol, DMSO, plant oils, triglyceride, silicone oils, and a wide-range of hydrophobic emollients. Hence, an oil-in-water type emulsion can be implemented to increase the bioavailability of bakuchiol via the skin. This type of emulsion (containing water) was used in cosmetic samples 5 and 6. The other products were squalene/oil/oil mixture solutions.

### 2.1. UV-Vis Analysis

All the examined samples and the standard were subjected to UV analysis in ethanol.

**Figure 2:**
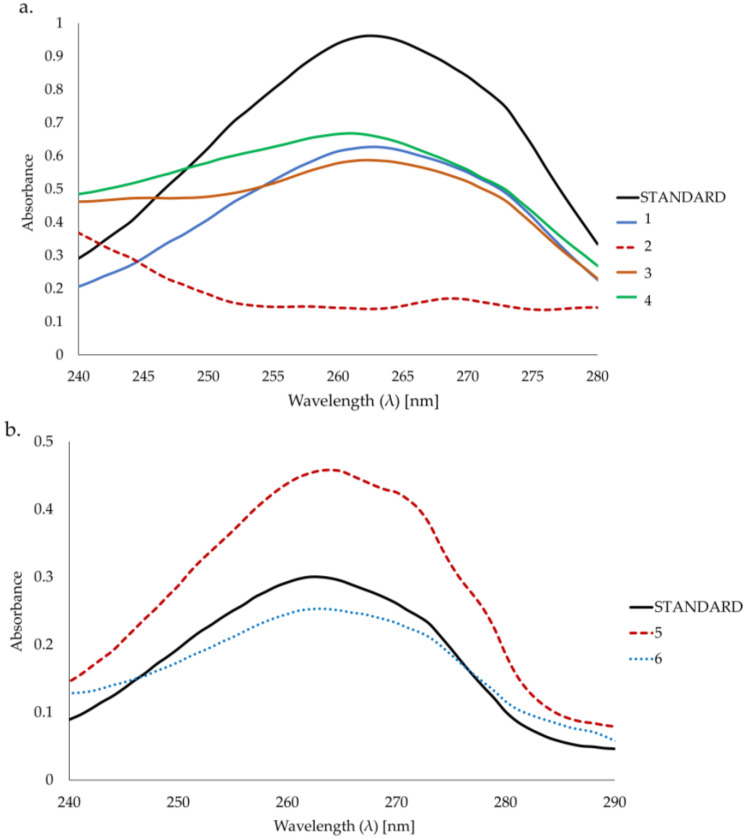
(**a**). UV-Vis spectra of bakuchiol (standard) (15 μg/mL) in samples 1, 2, 3, and 4. (**b**). UV-Vis spectra of bakuchiol (standard) (5 μg/mL) in samples 5 and 6.

The shape of the spectra in samples 1, 3, and 4 was similar to the standard (Figure 2a and Figure S1). Based on the UV-Vis spectrum, the wavelength of 262 nm was chosen for the quantitative determination of bakuchiol. The analysis of the UV-Vis spectra of sample 2 shows no presence of bakuchiol (Figure 2a). The content of bakuchiol was determined using a standard curve (see Section 4). 

Samples 5 and 6 could not be dissolved completely and bakuchiol could not be properly extracted because of the oil-in-water type of emulsion used; however, in the UV spectra of the solutions obtained from partial dissolution (Figure 2b and Figure S2), a maximum at λ = 262 nm is present and the shape of the spectra allows one to conclude that bakuchiol is probably present in the sample but could not be quantified.

### 2.2. HPLC Analysis

Bakuchiol content in cosmetic products was analyzed using the HPLC-DAD method. The components are well-separated using a reverse-phase column (endcapped C18) and an isocratic elution with acetonitrile containing 1% formic acid. In accordance with the UV spectra the detection wavelength was set at λ = 260 nm. The peak at RT 31.8 min was assigned to bakuchiol. The limit of detection (LOD) and limit of quantification (LOQ) were determined with the formulas LOD = 3.3 × σ/S and LOQ = 10 × σ/S, where σ is the standard deviation of the y-intercept and S is the slope of the calibration curve. The relative standard deviation (% RSD) for intraday variation remained below 2.5%.

**Figure 3:**
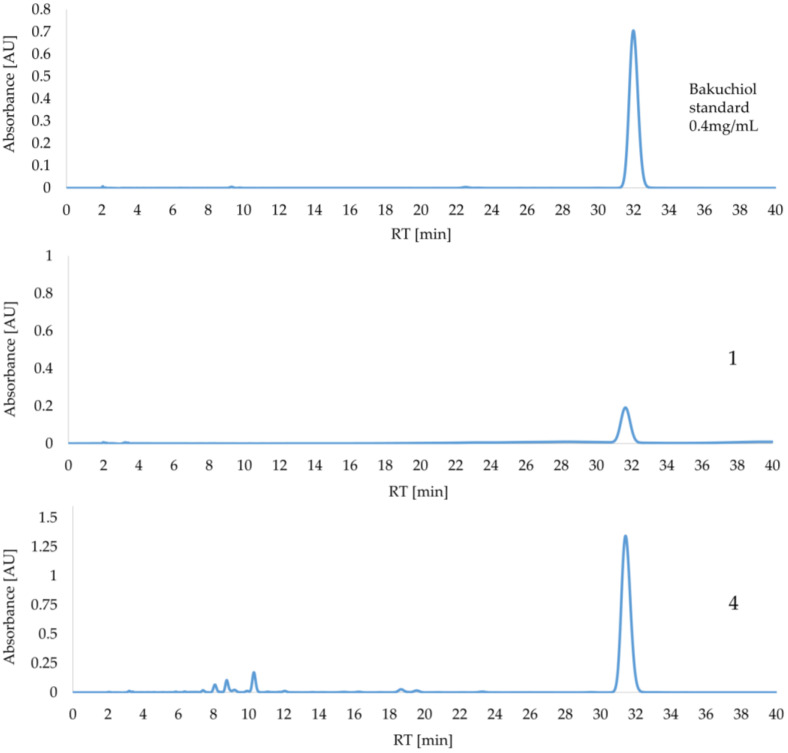
The chromatograms of bakuchiol standard (0.4 mg/mL) and samples 1 and 4 at λ = 260 nm.

Representative chromatograms of samples 1 and 4 are presented in Figure 3 (all obtained chromatograms are shown in Figure S3). The late-eluting bakuchiol content could be determined because no peak interference with the other ingredients was observed.

The HPLC findings correspond to the results of the UV/VIS method. Sample 4 had the highest bakuchiol content at 3.6%. Sample 3 matched the amount stated on the product label at 1%, whereas sample 1 contained only 50% of the declared content, namely 0.51%. No bakuchiol was detected in sample 2.

### 2.3. NMR Analysis

**Figure 4:**
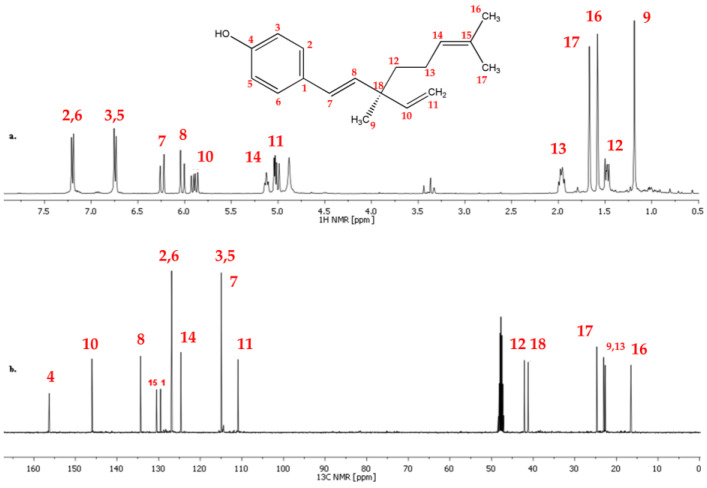
(**a**) ^1^H and (**b**) ^13^C NMR spectra of bakuchiol (standard) in CDCl_3_ (300 MHz) with atom assignment (red numbers).

Based on the recorded sets of spectra (^1^H, ^13^C, DEPT, COSY, HSQC, and HMBC) of bakuchiol (standard) (Figure 4 and Figures S4–S12 in Supplementary Materials), the structure was confirmed by assigning ^1^H and ^13^C signals. The ^1^H and ^13^C chemical shifts are summarized in Table 2 and the assignment is presented in Figure 4.

The ^1^H spectrum of bakuchiol measured in CDCl_3_ consists of characteristic signals of a *para*-substituted aromatic at δ = 7.25–7.20 ppm and δ = 6.80–6.70 ppm; the signals at δ = 6.30–6.20 ppm; δ = 6.10–6.00 ppm; δ = 5.95–5.85 ppm are typical of hydrogens attached to a double bond (Figure 4).

**Figure 5:**
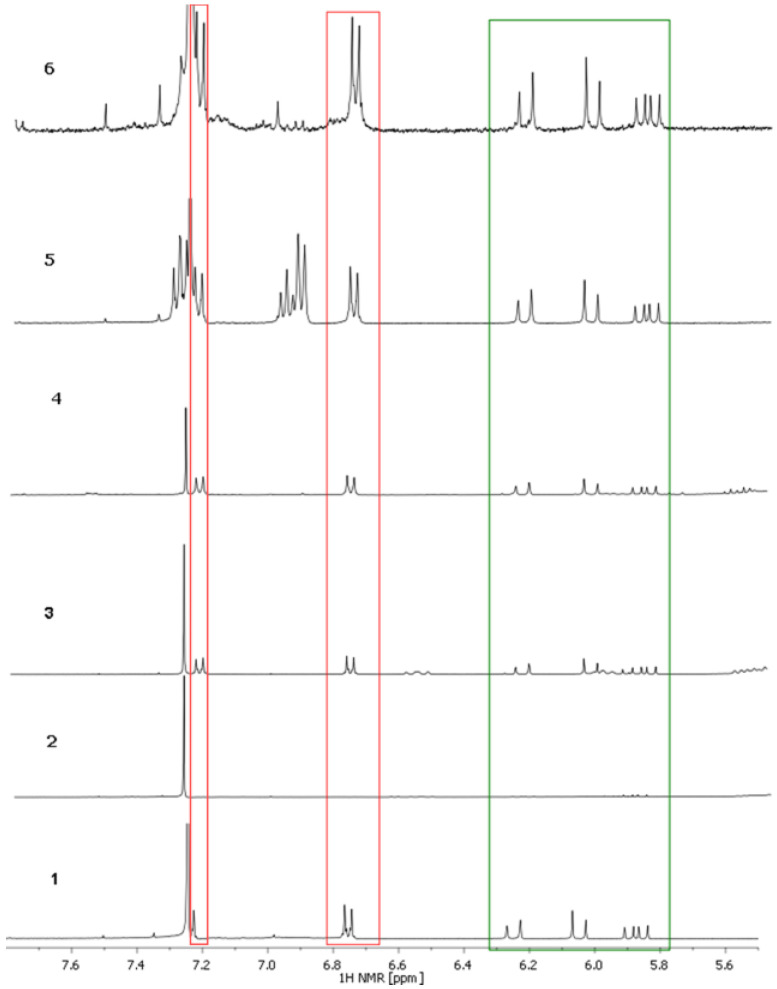
^1^H NMR spectra of the samples registered in CDCl_3_ (300 MHz). The signals of aromatic protons in bakuchiol are marked in red and the signals of hydrogens attached to double bonds are marked in green.

Since the cosmetic products contain multiple other components (cf. Table 1), the signals of bakuchiol are slightly shifted due to possible interactions. The spectra of the six samples are presented in Figure 5. The additional signals can be easily identified. They occur primarily in the aliphatic range (0–5.0 ppm; cf. also Figure S13 in Supplementary Materials). The hydrogen signals of the CH_2_ and CH_3_ groups present in unsaturated fatty acids (δ range 0.89–0.91 ppm) are observed for samples 1–5, and proton signals of the CH_2_ and CH_3_ groups of the squalene are present in samples 1–4 [20]. The signals at δ 1.5 ppm are protons from acyl chains (-OCO-CH_2_-CH_2_-) in oils. For instance, in samples 2–4, a multiplet at δ 2.3 ppm signifies the presence of the -OCO-CH_2_- groups derived from acyl chains in unsaturated fatty acids, whereas the multiplets at approximately δ 4.4 ppm indicate the presence of triacylglycerols in the product. Sample 6 shows a different spectrum in the analyzed range. The multiplet at approximately δ 4.7 ppm shows the presence of glycerin, a finding that is corroborated by the composition of the preparation (Table 1). In three of the six analyzed samples (1, 5, and 6), a singlet appears in the spectrum at approximately δ 2.0 ppm; its chemical shift value indicates that it is a signal from the hydroxyl proton of the alcohol.

Not all bakuchiol signals are visible. As was found in the HPLC chromatogram, in sample 2 no bakuchiol signal was observed, even though the manufacturer’s declaration stated that the substance was present. This could be due to the fact that the amount of bakuchiol is too low in this sample.

For quantification of bakuchiol in the samples, the signals above 5.5 ppm would be appropriate due to less interference with the other signals of bakuchiol and the signals of possible excipients in the samples.

**Figure 6:**
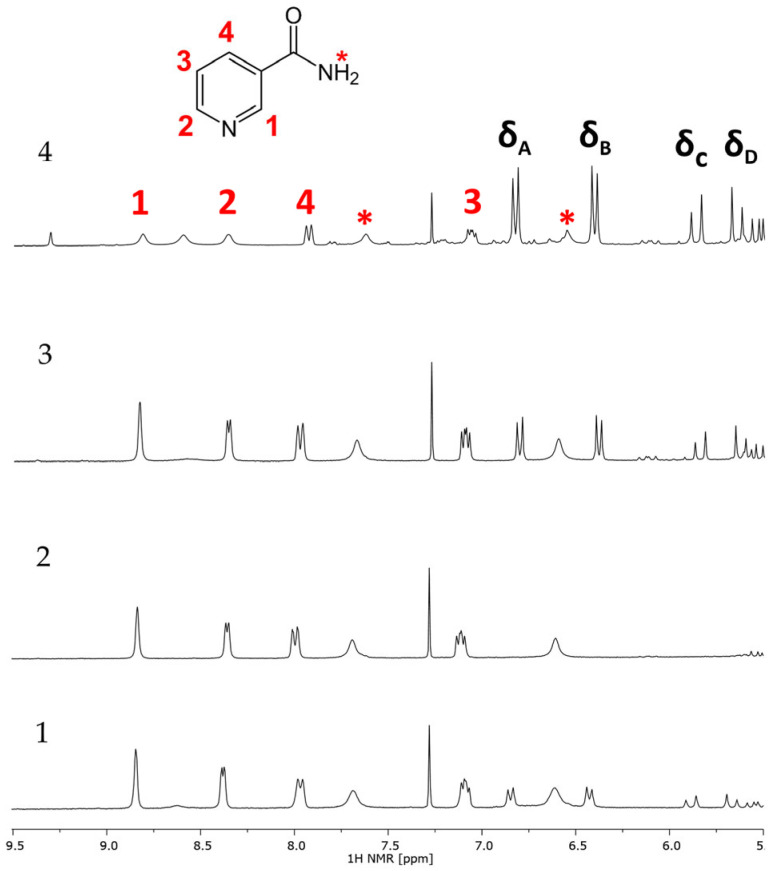
^1^H NMR spectra of samples 1, 2, 3, and 4 with nicotinamide added in CDCl_3_:DMSO (5:1, v:v) (300 Hz). The protons of nicotinamide are assigned in red; analytical signals of bakuchiol are assigned in black. The protons from the NH_2_ group are marked with a red asterisk.

Nicotinamide (niacinamide, vitamin B3, or vitamin PP) was selected as an internal standard in quantitative NMR due to its availability, lack of reactivity, similarity in solubility as well as its use in pharmaceutical and cosmetic applications (although not in the studied samples), its relatively simple structure, short acquisition time, simplicity of the signals, and the fact that in most cases the signals do not interfere with those of bakuchiol. The assignment of the nicotinamide signals in CDCl_3_ is presented in Figure S14 (Supplementary Materials). It is worth noting that in CDCl_3_ the signals of hydrogens of the amide group interfere with possible analytical signals of bakuchiol. This problem can be solved by using the CDCl_3_:DMSO (5:1, v:v) mixture as a solvent—the signals of nicotinamide and bakuchiol are well separated, as shown in Figure 6. As all the signals of nicotinamide are located in the aromatic region, the analysis could be focused in this region.

Based on the spectra of the samples recorded in the CDCl_3_:DMSO mixture, the analytical signals of bakuchiol were selected as δ_A_ = 6.88–6.78 ppm, δ_B_ = 6.46–6.36 ppm, δ_C_ = 5.94–5.80 ppm, and δ_D_ = 5.71–5.59 ppm.

The quantitative results obtained using the method with nicotinamide as an internal standard are presented in Table 3.

## 3. Discussion

The applied methods allowed for the confirmation or denial of bakuchiol content in the examined samples, as well as the quantification of bakuchiol in the samples soluble in the solvents used (Table 3).

Given that bakuchiol is insoluble in water but soluble in vegetable oils, triglycerides, and a wide range of hydrophobic emollients, an oil-in-water emulsion can be used in many preparations to increase the bioavailability of bakuchiol through the skin. This emulsion type was used in cosmetic samples 5 and 6, which were characterized by the presence of water. Consequently, direct determination (lacking additional extraction steps to separate the active substance) was not feasible. This encourages further analysis to optimize the extraction process for quantitative determination.

**Figure 7:**
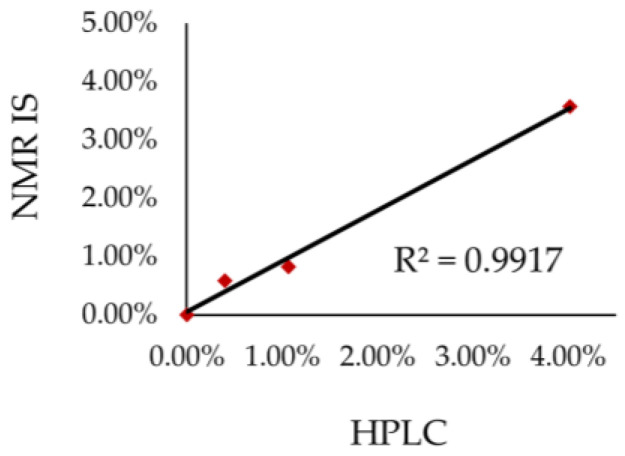
Correlation between the results obtained using ^1^H qNMR with internal standard.

The correlation between the results of both NMR and HPLC techniques (Figure 7) determined using linear regression indicates that the analytical results are fully convergent and thus can be successfully used interchangeably. The coefficients of determination R^2^ is close to 1 (0.9917). Hence, the qNMR method is competitive with the HPLC method. However, with regard to the analysis time, the NMR method is superior to HPLC. The analysis using the HPLC method takes about 40 minutes per sample, whereas in the case of NMR the time of analysis is much shorter, of about 4 minutes only.

The results of this study clearly demonstrate the necessity of examining the quality of cosmetic products and the quantification of the most important ingredient, in this case bakuchiol. Moreover, the results allow one to suspect that there is non-compliance with the manufacturer’s declaration in sample 2, in which bakuchiol content was not confirmed by any of the selective qualitative analysis methods. Likewise, the declared content of 1% bakuchiol in sample 1 could not be confirmed by any of the applied methods, with the results suggesting a content of approximately 0.5–0.6%. For sample 3, the obtained results were consistent with the manufacturer’s declaration, i.e., approximately 1% of bakuchiol content, whereas for sample 4, which had no declaration concerning bakuchiol content in the product label, the result obtained indicates that it contains approximately 4% of the determined meroterpene.

## 4. Materials and Methods

### 4.1. Materials

Bakuchiol (Standard) was obtained from Sigma Aldrich (CAS 10309-37-2). Nicotinamide (CAS 98-92-0) was purchased from Merck Polska (Merck KGaA, Darmstadt, Germany). The solvents methanol (CAS 67-56-1), ethanol (CAS 64-17-5), isopropanol (CAS 67-63-0), CDCl_3_ (CAS 865-49-6), DMSO (CAS 67-68-5, HCOOH (CAS 64-18-6), and acetonitrile (CAS 75-05-8) were obtained from Sigma Aldrich (Steinheim, Germany).

During solubility tests, samples 5 and 6 in the form of water-containing emulsion were excluded from the investigations, thus limiting the use of organic solvents such as chloroform (CDCl_3_).

### 4.2. Methods

#### 4.2.1. UV-Vis

The spectra were recorded using a UV/Vis Thermo Scientific Evolution 60S spectrometer, in the range of wavelength 200–300 nm, with ethanol as a solvent. Based on standard spectra, the wavelength of 262 nm was selected for quantification, in agreement with the literature [21]. A series of bakuchiol standard concentrations were prepared in the range of 5–20 μg/mL. Based on the measured absorbance, a calibration curve was obtained (Table 4).

Then, 25 mg of each sample was dissolved in 1.5 mL of ethanol and diluted. The concentration of each sample was calculated using the calibration curve.

#### 4.2.2. HPLC

Qualitative and quantitative analyses were performed using a Chromaster HPLC system (Hitachi, Tokyo, Japan) equipped with a gradient pump, a diode-array detector, an autosampler with a thermostat, and a column thermostat. A Merck A Purospher STAR RP-18e (5 µm, 250 mm × 4.6 mm) column was used with a flow rate of 1 mL/min at the temperature of 35 °C. The mobile phase consists of 1% formic acid and acetonitrile, 35/65 (v/v); the analysis was carried out under isocratic conditions. The injected volume was 20 µL, wavelength range 220–380 nm, and detection at λ = 260 nm.

A stock solution of bakuchiol (standard), 10 mg/mL in 2:1 (v:v) methanol/isopropanol, was prepared, and dilutions were used to obtain a calibration curve (Table 5).

For quantitative analysis, 20–30 mg of each sample was dissolved in 1.5 mL of 2:1 (v:v) methanol/isopropanol. The chromatograms were recorded at 260 nm. Bakuchiol content was calculated using the calibration curve.

#### 4.2.3. ^1^H and ^13^C NMR

The ^1^H and ^13^C NMR spectra (Figure 4), as well as the 2D experiments COSY, HMQC (Figure S1), HSQC, and HMBC were recorded using a VARIAN VNMRS spectrometer operating at 299.61 (^1^H) and 75.13 (^13^C) MHz, with a gradient probe (AutoSw PFG 4 Nuc type, 5 mm) direct type with an axis gradient. The used frequency was 100 MHz. SW = 16 ppm, t_aq_ = 2.05 s, delay D_1_ = 1 s, and NS = 64. NMR tubes of 5 mm were used with CDCl_3_ as solvent.

Based on the spectra of bakuchiol (standard), the signals of bakuchiol were assigned. The samples were dissolved in CDCl_3_ (samples 5 and 6 only partially) and the ^1^H spectra were recorded (Figure 5 and Figure S2 in Supplementary Materials).

#### 4.2.4. qNMR Internal Standard (IS)

Nicotinamide was chosen as the internal standard as it would minimally interfere with the analytical signals of bakuchiol and the signals from other components (oils). First, 250 mg of each sample was dissolved in 1 mL of 5:1 (v:v) CDCl_3_:DMSO and 800 µL of each of the obtained solutions was placed in NMR tubes. Then, 50 µL of the nicotinamide standard solution (45.73 mg/mL) was added to every tube. Each sample was stirred and the ^1^H spectra were recorded. After FT and baseline correction, all analytical signals of bakuchiol (δ_A_ = 6.88–6.78 ppm; δ_B_ = 6.46–6.36 ppm; δ_C_ = 5.94–5.80 ppm; δ_D_ = 5.71–5.59 ppm) and nicotinamide (δ_IS1_ = 8.88–8.75 ppm; δ_IS2_ = 8.42–8.30 ppm; δ_IS4_ = 8.04–7.89 ppm; δ_IS3_ = 7.15–7.02 ppm) that did not interfere with any other signal were manually integrated. Only in sample 1 the integration of all signals was possible. In sample 3, the δ_D_ signal interfered with the signal from other ingredients contained in the sample, which was also the case in sample 4, with the signals δ_A,_ δ_D,_ δ_IS1_, and δ_IS3_ interfering.

Using the area under the curve of all integrated signals, the concentration of each sample was calculated with the following formula:

$$\%=\frac{I_{BAK}}{I_{IS}}\;*\;\frac{n_{IS}}{n_{BAK}}\;*\;\frac{M_{BAK}}{M_{IS}}\;*\;\frac{m_{IS}}{m_{BAK}}\;*\;P,$$

where I_BAK_ and I_IS_ are integrities of bakuchiol and internal standard (nicotinamide) signals, respectively; n_IS_ and n_BAK_ are numbers of equivalent protons producing the integrated signals; M_BAK_ and M_IS_ are molar masses of the examined compounds; m_IS_ and m_BAK_ are the mass of internal standard and bakuchiol, respectively in the NMR tube, and P is the purity of internal standard used.

## 5. Conclusions

Because of the difficulty of working with this type of research material (complex mixtures—cosmetic serum) as well as a wide variety of potential samples, it might be valuable to consider developing a method for extracting bakuchiol (an active substance which has a partially terpenoid structure) in order to extend the possibilities of using the methods presented. Thus, it would be possible to include a much more diverse material both in composition and form, and also to increase the reliability of the obtained results. However, the principal aim of this study was to assess the usefulness of qualitative and quantitative analysis methods by which the identity of bakuchiol could be confirmed and quantified in a complex material such as serum-type cosmetics in a relatively short time and at low cost. UV-Vis spectrophotometry, NMR spectroscopy, and HPLC chromatography were successfully employed to unambiguously assess the quality and verify the manufacturer’s declarations of selected cosmetic products with diversified compositions and prices available on the Polish market.

The present study demonstrated that the composition of cosmetic products can prove challenging to analyze. Samples 5 and 6 contained bakuchiol, but were biphasic systems. In this case, it was impossible to analyze the product directly, which would make it necessary to search for an alternative procedure. One potential method that was planned for use in future studies was lyophilization of the emulsion [22], followed by determination by liquid or gas chromatography. In the present study, the HPLC method was used; however, gas chromatography could also be applied [23]. This would obviate the need for an extraction step, enabling the bakuchiol content to be determined using a small amount of sample processing. It is also conceivable to use a direct determination method derived from nanoemulsion products [24].

## Figures and Tables

**Table 1:** Declared composition of the studied cosmetic products.

Sample	Declared Bakuchiol Content	Ingredients (INCI)
1	1%	Squalane, Bakuchiol
2	No declaration	Caprylic/Capric Triglyceride, Coco-Caprylate/Caprate, Helianthus Annuus (Sunflower) Seed Oil, Squalane, Bakuchiol, Bacillus Ferment Lysate, Mangifera Indica (Mango) Seed Butter, Tocopherol, Parfum, Citral, Citronellol, Geraniol, Limonene, Linalool
3	1%	Argania Spinosa Kernel Oil, Prunus Amygdalus Dulcis Oil, Vitis Vinifera Seed Oil, Caprylic/Capric Triglycerides, Squalane, Coco-Caprylate, Bakuchiol, Isoamyl Laurate, Isoamyl Cocoate, Oenothera Biennis Seed Oil, Carum Petroselinum Seed Oil, Rosa Canina (Fruit) Oil, Simmondsia Chinensis Seed Oil, Rubus Idaeus Seed Oil
4	No declaration	Squalane (Olive), Alpha Lipoic Acid, Bakuchiol, Geraniol, Linalool, Citral, Limonene
5	1%	Aqua, Propanediol, Isopropyl myristate, Caprylic/capric triglyceride, Vitis vinifera seed oil, Bakuchiol, Phenoxyethanol, Polyacrylamide, Xanthan gum, C13–14 isoparaffin, Titanium dioxide, Laureth-7, Ethylhexylglycerin, Ci 17200 (red 33), Ci 42090 (blue 1)
6	No declaration	Aqua, Rosa damascena flower water, Alcohol, Xanthan gum, Glycerin, Bakuchiol, Parfum, Citric acid, Benzyl salicylate, Tartaric acid, Lactic acid, Citronellol, Limonene, Geraniol

**Table 2:** Assignment of signals in ^1^H and ^13^C NMR spectra of bakuchiol (standard).

	δ ^1^H [ppm]	δ ^13^C [ppm]
1	-	129
2	7.20	126
3	6.74	115
4	-	155
5	6.74	115
6	7.20	126
7	6.24	126
8	6.02	134
9	1.19	22
10	5.90	146
11	5.03	111
12	1.48	41
13	1.97	23
14	5.12	124
15	-	130
16	1.58	16
17	1.68	25
18	-	42

**Table 3:** Bakuchiol content in the products obtained by NMR-IS, UV/VIS, and HPLC-DAD methods, percentage [% w/w] ± SD; ND—not detected.

	Declared Bakuchiol Content	Result ± SD		
Sample		NMR-IS	UV-Vis	HPLC-DAD
1	1%	0.59% ± 0.06	0.55% ± 0.001	0.51 ± 0.01
2	No declaration	ND	0.17% ± 0.001	ND
3	1%	0.83% ± 0.01	1.04% ± 0.001	1.06 ± 0.01
4	No declaration	3.57% ± 0.21	3.99% ± 0.004	3.62 ± 0.01
5	1%	-	0.45% ± 0.001	-
6	No declaration	-	0.23% ± 0.001	-

**Table 4:** Validation parameters for the UV-Vis method.

Calibration Curve	R^2^	Linear Range (µg/mL)	LOD (µg/mL)	LOQ (µg/mL)
A = 0.0699 C − 0.0724	0.999	5–30	0.88	2.67

**Table 5:** Concentration range, limit of detection (LOD), and limit of quantification (LOQ) parameters obtained by the HPLC-DAD method.

Calibration Curve	R^2^	RT [min]	Linear Range (mg/mL)	LOD (mg/mL)	LOQ (mg/mL)
A = 45,717,172 C − 6656	0.997	31.8	0.05–0.5	0.023	0.069

## Data Availability

The original contributions presented in this study are included in the article/Supplementary Material. Further inquiries can be directed to the corresponding author.

## Supplementary Materials

The following supporting information can be downloaded at: https://www.mdpi.com/article/10.3390/ijms26146638/s1.
