# Supplementary material for: Comparison of Quantification Using UV-Vis, NMR, and HPLC Methods of Retinol-Like Bakuchiol Present in Cosmetic Products
Source: Int J Mol Sci. 2025 Jul 10;26(14):6638. doi: 10.3390/ijms26146638 (PMC12295351; doi:10.3390/ijms26146638)
Supplement: Supplementary file 1 [file ijms-26-06638-s001.zip › ijms-3674611-supplementary.pdf]

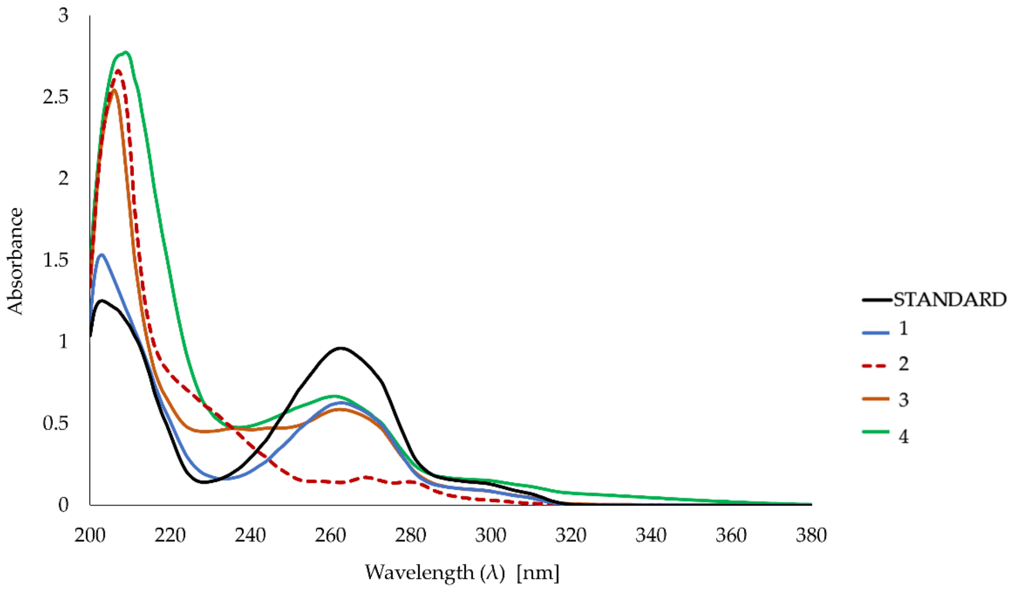

Figure S1. UV-Vis spectra of bakuchiol standard (15µg/mL) and samples 1-4 (200-380nm).

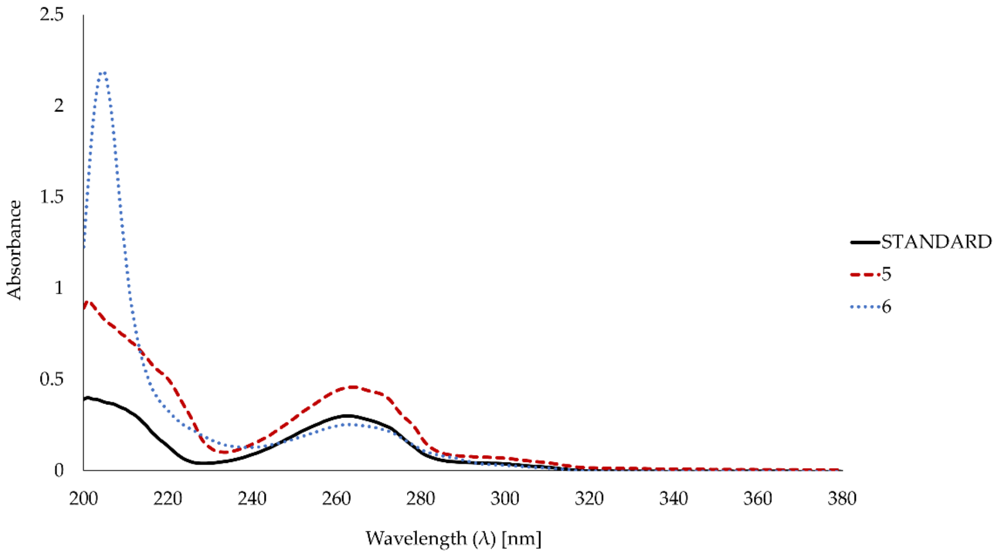

Figure S2. UV-Vis spectra of bakuchiol standard (5µg/mL) and samples 5 and 6 (200-380nm).

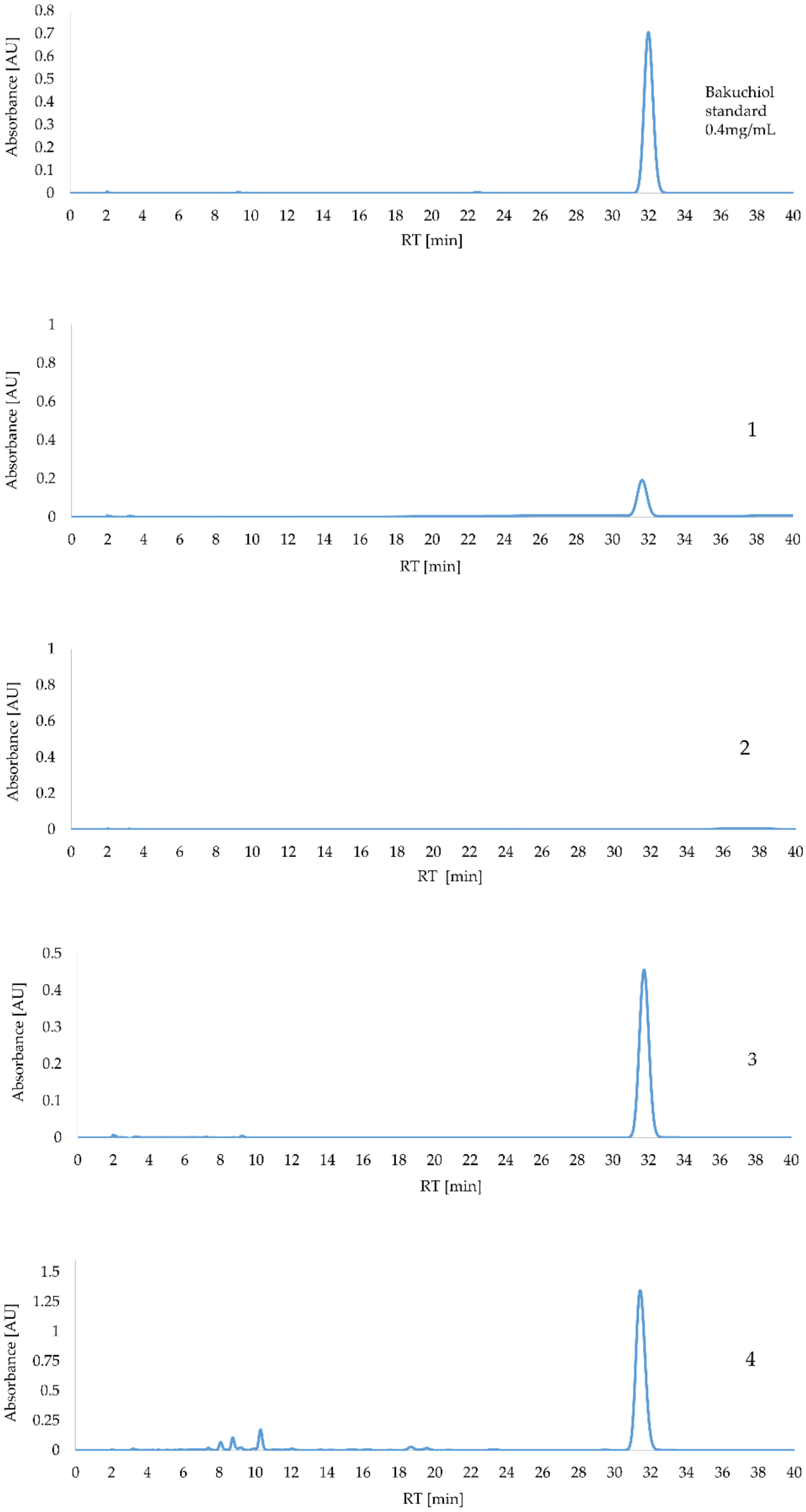

Figure S3. The chromatograms of bakuchiol standard (0.4mg/mL) and samples 1-4 at λ = 260 nm.

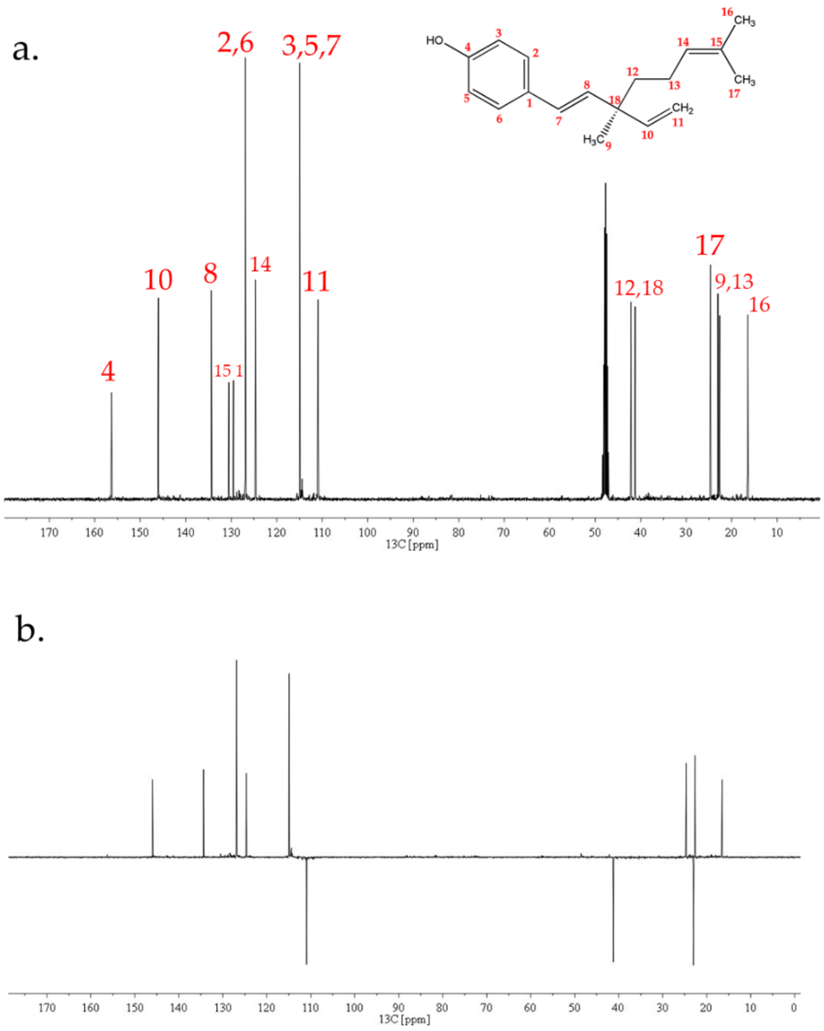

Figure S4. a. ^{13}C NMR spectrum of bakuchiol standard b. DEPT-135 ^{13}C NMR spectrum of bakuchiol standard.

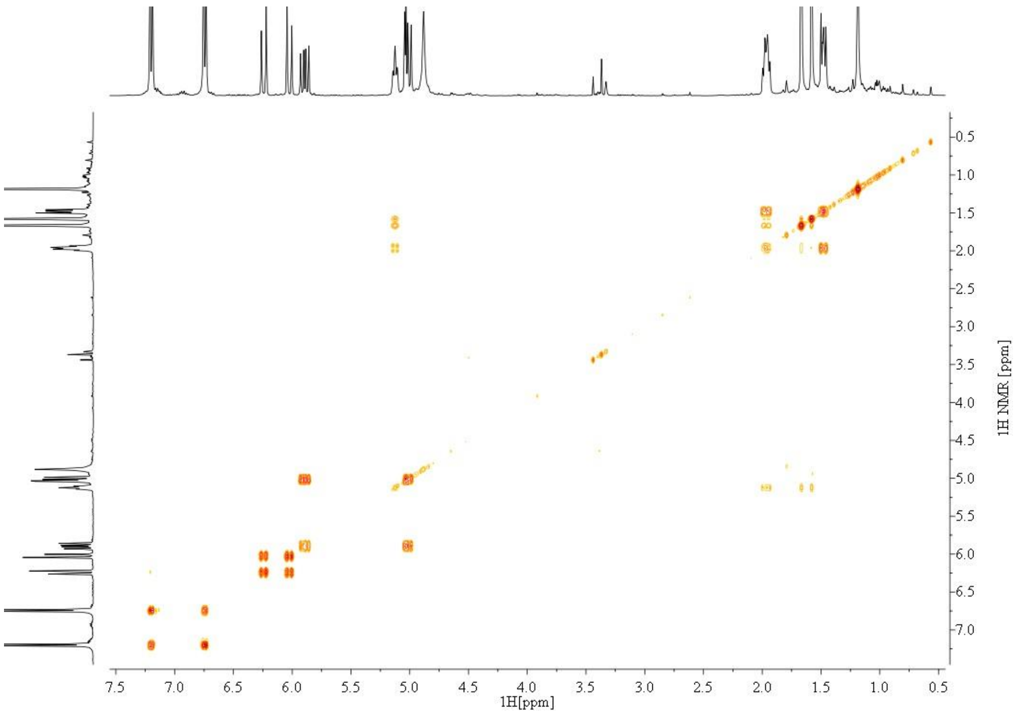

Figure S5. COSY ^1H NMR correlation spectrum of bakuchiol standard in $CDCl_3$. (300MHz).

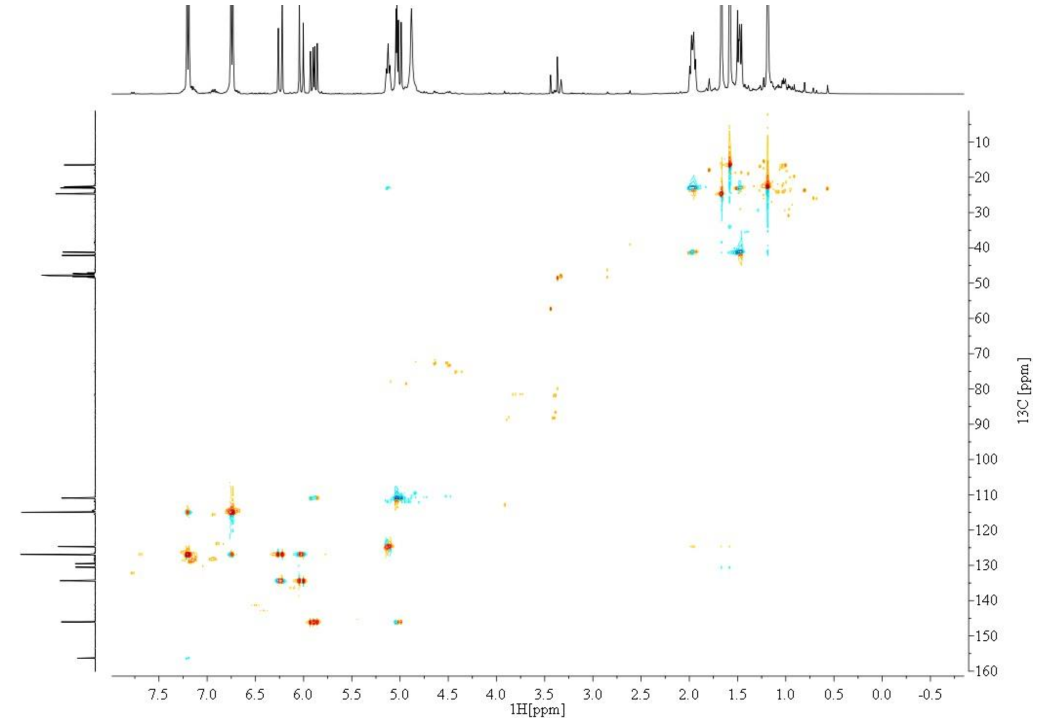

Figure S6. HSQC ^1H-^{13}C NMR correlation spectrum of bakuchiol standard in $CDCl_3$. (300MHz).

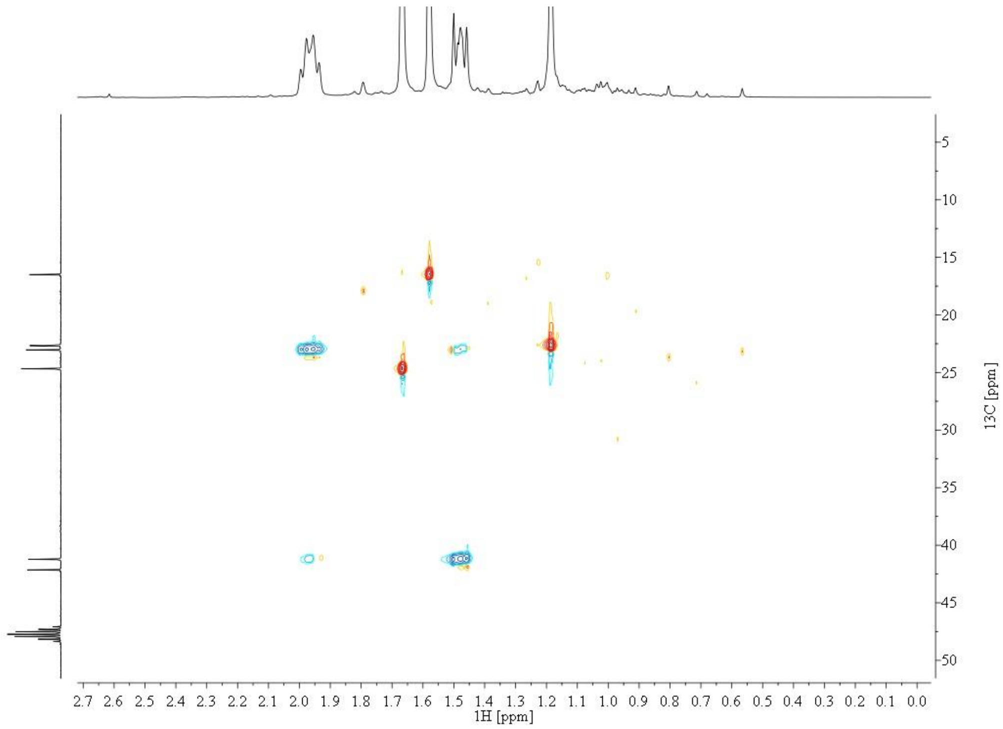

Figure S7. HSQC ^{1}H-^{13}C NMR correlation spectrum of bakuchiol standard in $CDCl_3$. (300MHz). (^{1}H: 2.7-0ppm; ^{13}C: 50-0ppm)

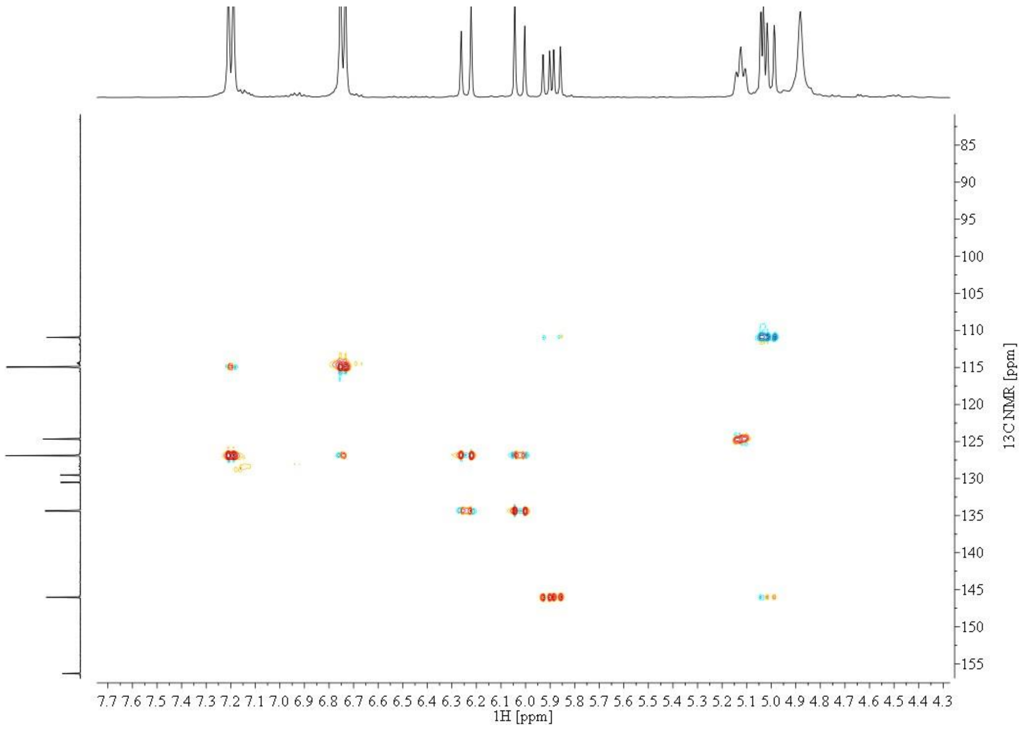

Figure S8. HSQC ^{1}H-^{13}C NMR correlation spectrum of bakuchiol standard in $CDCl_3$. (300MHz). (^{1}H: 7.7-4.3ppm; ^{13}C: 155-80ppm)

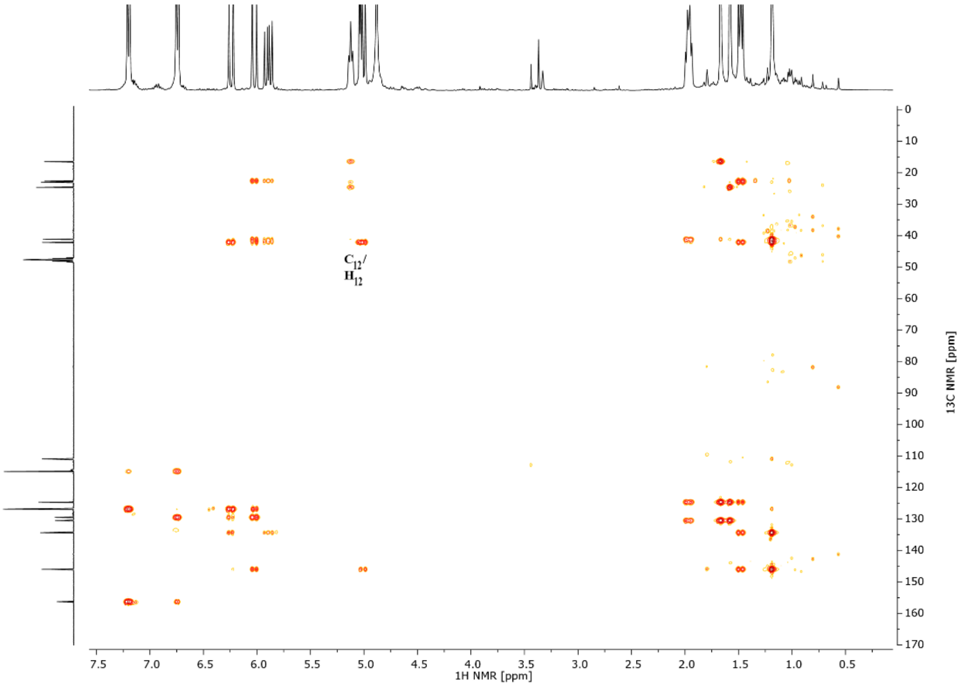

Figure S9. HMQC ^1H-^{13}C NMR correlation spectrum of bakuchiol standard in $CDCl_3$. (300MHz)

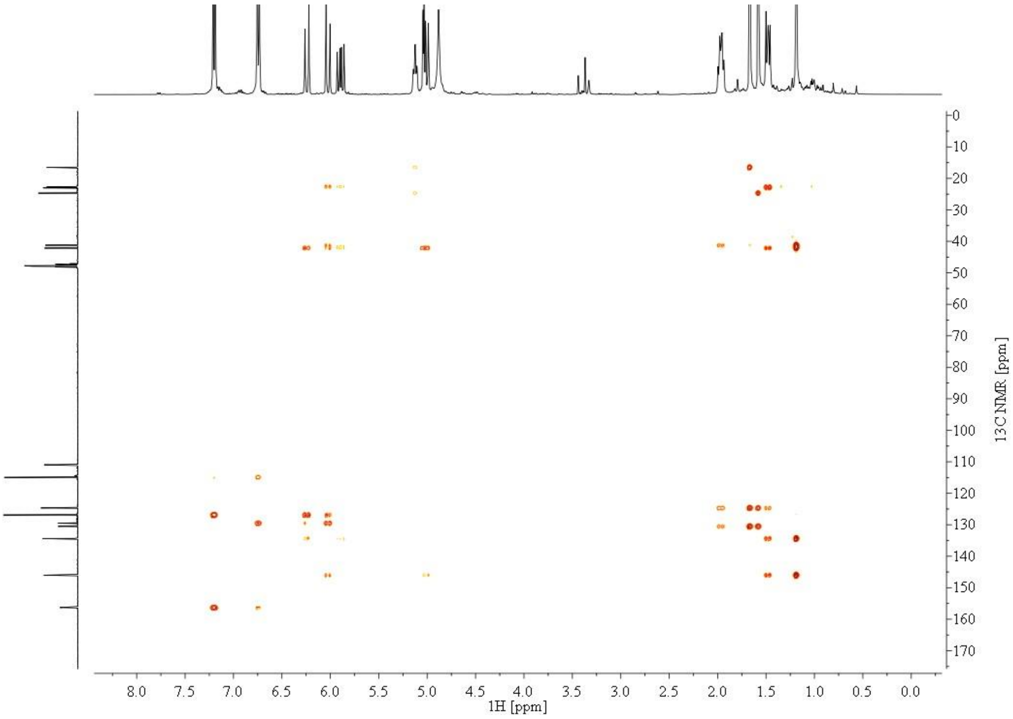

Figure S10. HMBC ^1H-^{13}C NMR correlation spectrum of bakuchiol standard in $CDCl_3$. (300MHz).

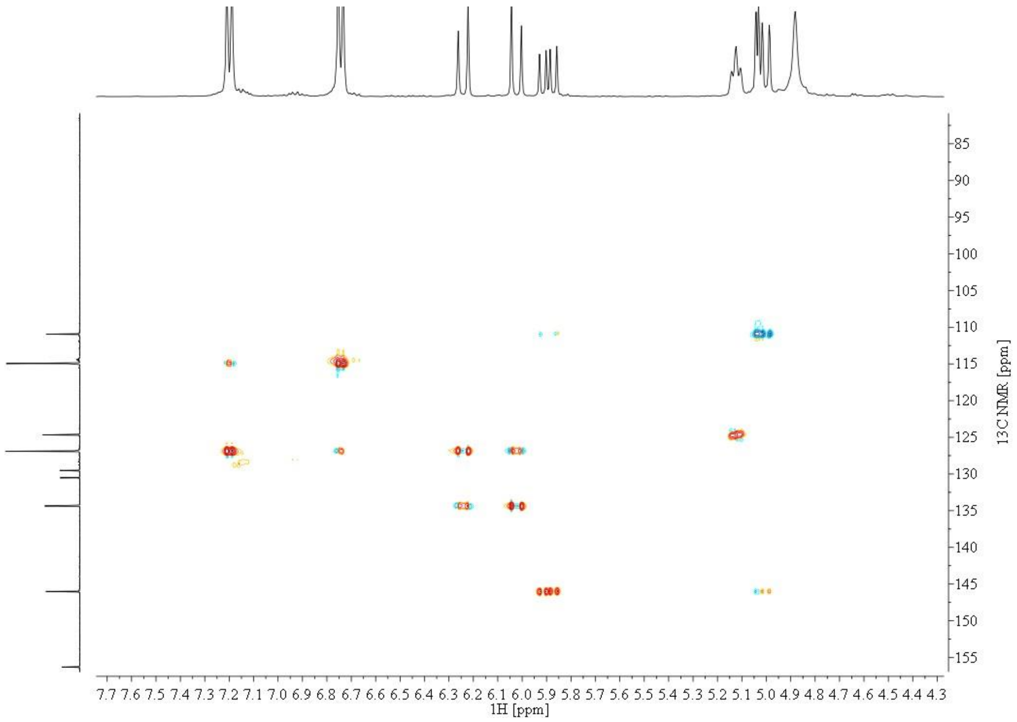

Figure S11. HMBC ^{1}H-^{13}C NMR correlation spectrum of bakuchiol standard in $CDCl_3$. (300MHz). (^{1}H: 7.7-4.3ppm; ^{13}C: 155-80ppm)

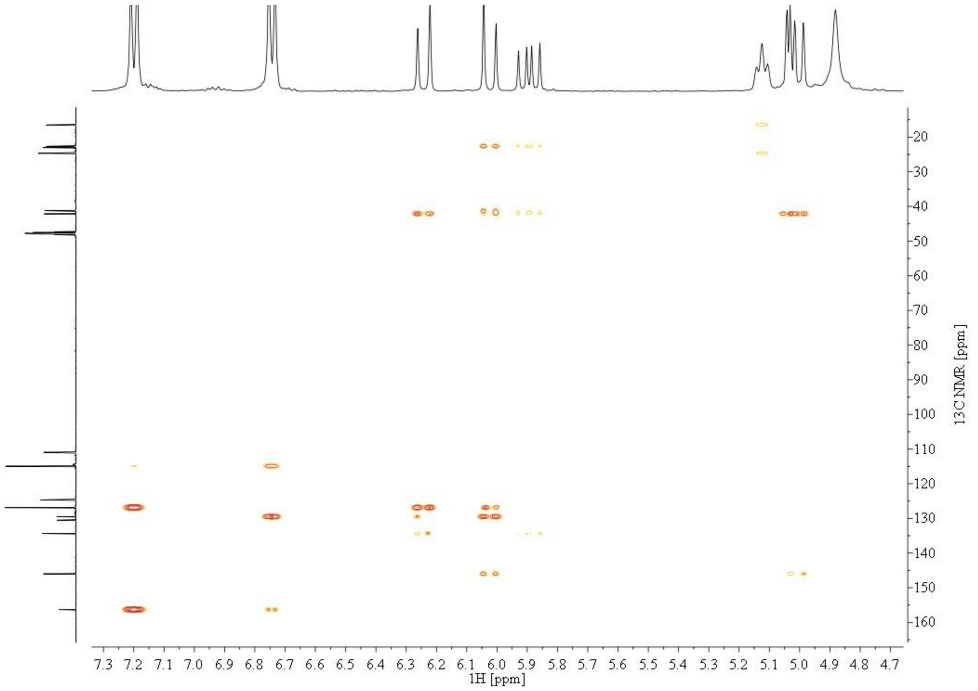

Figure S12. HMBC ^{1}H-^{13}C NMR correlation spectrum of bakuchiol standard in $CDCl_3$. (300MHz). (^{1}H: 7.3-4.7ppm; ^{13}C: 160-10ppm)

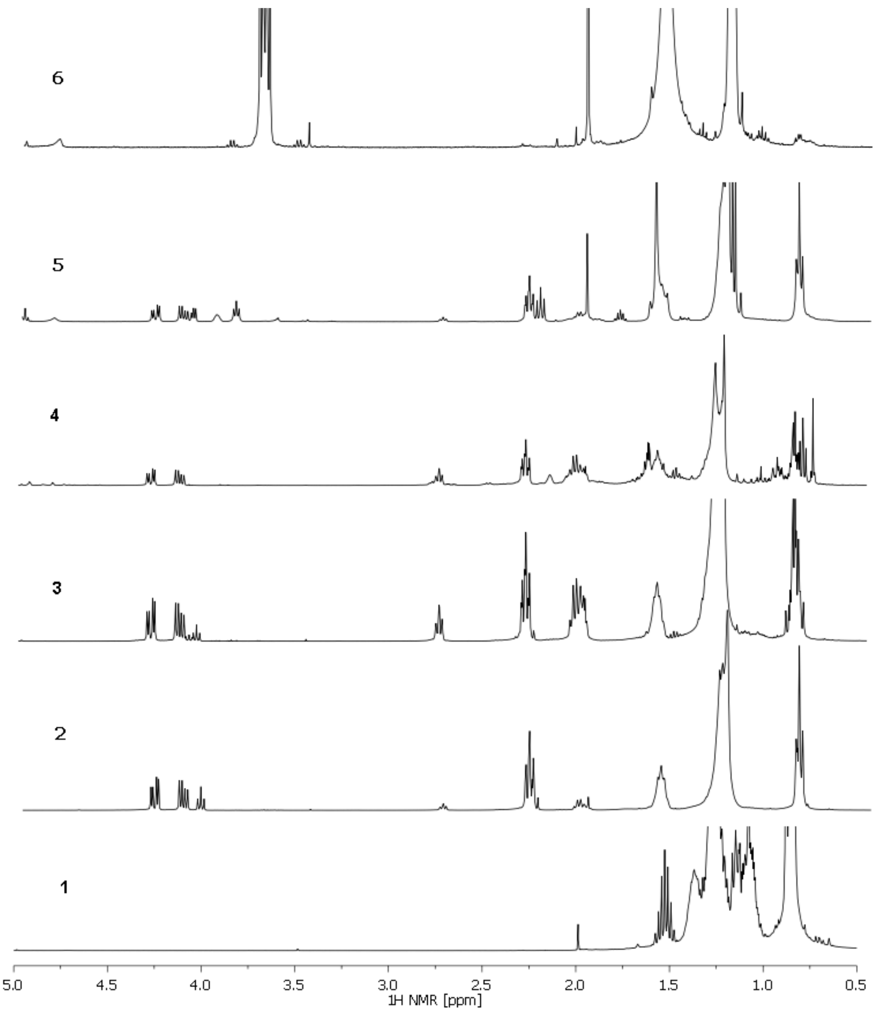

Figure S13. ^{1}H NMR spectra of samples 1-6. (5.0-0.5ppm) in $CDCl_3$. (300MHz)

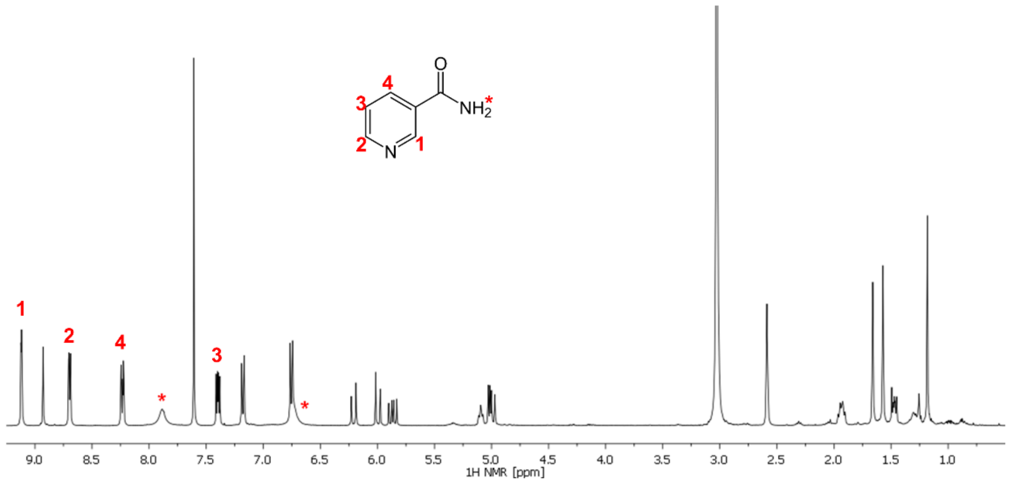

Figure S14. ^{1}H NMR spectrum of bakuchiol and nicotinamide in $CDCl_3$. (300MHz) Protons of nicotinamide are assigned in red.
